# Autoimmune Encephalitis in COVID-19 Infection: Our Experience and Systematic Review of the Literature

**DOI:** 10.3390/biomedicines10040774

**Published:** 2022-03-25

**Authors:** Adina Stoian, Mircea Stoian, Zoltan Bajko, Smaranda Maier, Sebastian Andone, Roxana Adriana Cioflinc, Anca Motataianu, Laura Barcutean, Rodica Balasa

**Affiliations:** 1 Department of Pathophysiology, George Emil Palade University of Medicine, Pharmacy, Sciences and Technology Targu Mures, 540136 Targu Mures, Romania; adina.stoian@umfst.ro; 2 Ist Neurology Clinic, Mures County Clinical Emergency Hospital, 540136 Targu Mures, Romania; smaranda.maier@umfst.ro (S.M.); adn.sebastian.007@gmail.com (S.A.); roxana.cioflinc@yahoo.ro (R.A.C.); laura.barcutean@umfst.ro (L.B.); rodica.balasa@umfst.ro (R.B.); 3 Department of Anesthesiology and Critical Care Medicine, George Emil Palade University of Medicine, Pharmacy, Sciences and Technology Targu Mures, 540136 Targu Mures, Romania; mircea.stoian@umfst.ro; 4 Department of Neurology, George Emil Palade University of Medicine, Pharmacy, Sciences and Technology Targu Mures, 540136 Targu Mures, Romania

**Keywords:** COVID-19 infection, autoimmune encephalitis, voltage-gated potassium channels antibody

## Abstract

The neurologic complications of COVID-19 infection are frequent in hospitalized patients; a high percentage of them present neurologic manifestations at some point during the course of their disease. Headache, muscle pain, encephalopathy and dizziness are among the most common complications. Encephalitis is an inflammatory condition with many etiologies. There are several forms of encephalitis associated with antibodies against intracellular neuronal proteins, cell surfaces or synaptic proteins, referred to as autoimmune encephalitis. Several case reports published in the literature document autoimmune encephalitis cases triggered by COVID-19 infection. Our paper first presents our experience in this issue and then systematically reviews the literature on autoimmune encephalitis that developed in the background of SARS-CoV-2 infections and also discusses the possible pathophysiological mechanisms of auto-immune-mediated damage to the nervous system. This review contributes to improve the management and prognosis of COVID-19-related autoimmune encephalitis.

## 1. Introduction

Coronavirus disease (COVID-19) is on ongoing pandemic with more than 298 million confirmed cases and more than 5.4 million deaths reported by the World Health Organization (covid19.who.int) on 7 January 2022, with a clinical spectrum of disease ranging from asymptomatic/mild to fatal disease [1].

The severe acute respiratory syndrome coronavirus 2 (SARS-CoV-2, the coronavirus that causes COVID-19) pandemic has had a severe impact on socio-economic and public health systems worldwide with few comparable events in the contemporary era. At the beginning of the pandemic, the challenge was represented by acute and dramatic outcomes, but over time, longer-term consequences and manifestations have become evident [2].

With the onset of SARS-CoV-2 infection, complications affecting multiple systems and organs have been described, including the involvement of the central nervous system (CNS) and the peripheral nervous system (PNS) [3,4,5,6], although the neurotropic potential of SARS-CoV-2 infection is still under debate. Infections with other coronaviruses have the potential for targeting the CNS, and neurological symptoms including headache, delirium, and anosmia have been described in up to 30% of COVID-19 patients in epidemiological studies [3,7,8,9,10]. Mao et al. published a study in 2020 from China in which neurological manifestations such as dizziness, headache, delirium and alterations of consciousness were present in up to 36.4% of patients hospitalized with COVID-19 [10]. The ability of the virus to function as a trigger of some autoimmune diseases raised in described cases of autoimmune rheumatic diseases, cases of myelitis, and Guillain-Barré Syndrome (GBS) that started after COVID-19 [5,11,12]. There were also case reports of autoimmune encephalitis, some in which the presence of autoantibodies was highlighted and other cases in which this diagnosis was assumed as a result of the clinical, imaging investigations and after the evolution under immunomodulatory therapy [4,13,14].

Little is known at the moment about the immune-mediated neurological diseases of the CNS following SARS-CoV-2 infection. In most of the cases with severe COVID-19 and neurological involvement, no evidence of corticospinal fluid (CSF) infection with SARS-CoV-2 was reported [15,16,17], and only a few reported cases showed a positive SARS-CoV-2 PCR test in CSF [18,19].

In this article, we present the case of a patient with a history of SARS-CoV-2 mild infection who was diagnosed at an interval of 4 months with autoimmune encephalitis. We conducted a literature research of other reported cases in which autoantibodies were evidenced to emphasize the possibility of an autoimmune process triggered by SARS-CoV-2 infection.

## 2. Materials and Methods

We performed a systematic review of the literature following Preferred Reporting Items for Systematic Review and Meta-Analyses (PRISMA) guidelines (http://www.prisma-statement.org/, accessed on 4 January 2022) using articles from the National Center for Biomedical Information-PubMed/Medline journal data base following search terms: “COVID-19” or “SARS-CoV-2” and “autoimmune encephalitis.” All articles with relevant titles were analyzed and screened for a possible association between COVID-19 and autoimmune encephalitis. The analyzed articles were published between 1 January 2020 and 31 December 2021. Searches were performed on 4 January 2022. A total of 154 articles met the criteria according to the search words and treated subjects. The reference list of the found articles was also screened and analyzed for additional case reports or case series related to autoimmune encephalitis, and another 21 articles were discovered.

**Figure 1:**
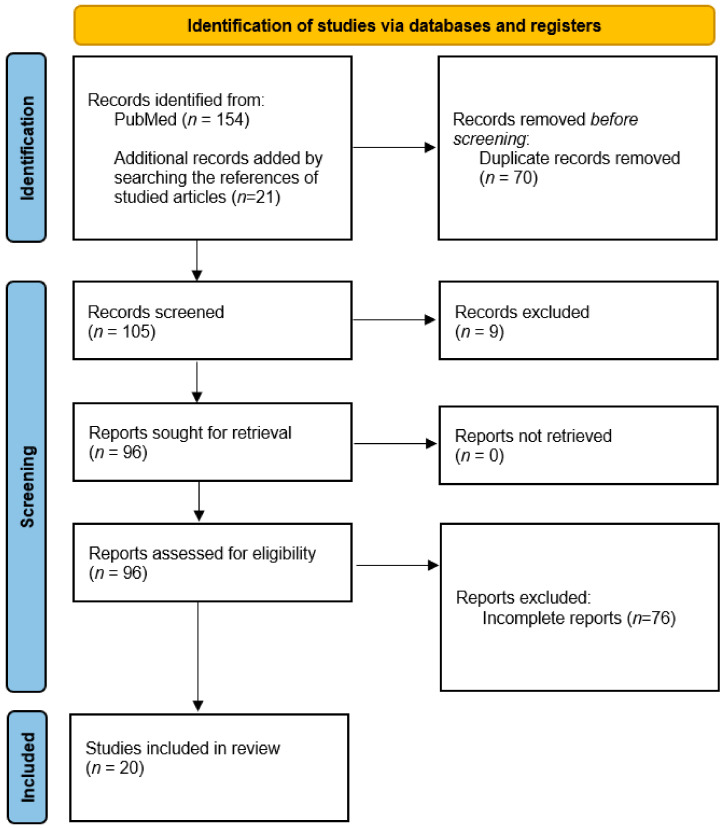
PRISMA flow diagram for the systematic review.

We removed the duplicates (*n* = 70), and 105 records were screened and analyzed, after which 20 publications fulfilled the inclusion and exclusion criteria summarized below. A flow chart of the search strategy is represented in Figure 1.

*Inclusion criteria*: We included studies or case series of patients diagnosed with COVID-19, with confirmed autoimmune encephalitis according to typical diagnostic criteria. In the end, we kept only those articles in which the presence of autoantibodies was identified in the serum or CSF and had a clear description of the case. Only papers in English were considered. All titles and articles were selected and extracted by two different experts that worked independently.

*Exclusion criteria*: The exclusions criteria were non-English articles, no human subjects, general reviews, other neurologic sufferance than autoimmune encephalitis and no clinical characteristics and autoantibodies reported.

Data extracted from the selected articles were age, gender, method of diagnosis (presence of RT-PCR swab test, brain imagistic results, EEG, and autoantibodies), general and neurological symptoms, the time intervals between the COVID-19 and autoimmune disease onset, treatments and outcomes. 

## 3. Results

### 3.1. Our clinical experience

An interesting case of a 67-year-old woman patient was admitted to the Neurology clinic in April 2021 due to disorientation, reduced verbal fluency, aphasia, confusion, ataxia, visual hallucinations, anxiety and disturbing insomnia with progressive evolution in the last 10 days before hospitalization. According to relatives, the symptoms started 2 or 3 months earlier with mild cognitive disorders, a depressive mood in the context of mourning (death of her husband due to COVID-19 3 months before) and asthenia. In the previous week before admission, she began to present with mild confusion, insomnia, ideo-motor slowing, reduced communication, cognitive fluctuations, memory troubles and slight disorientation with progressive evolution.

At admission, she presented with irritability and truncal ataxia that made walking and sitting impossible. She also presented with motor aphasia apraxia and difficulties performing simple commands. She appeared confused and disoriented in time and space and was unable to have a simple conversation. From her past medical history, we found that she suffered from increased blood pressure values, chronic atrial fibrillation, diabetes mellitus type 2 and dyslipidemia. Four months prior to the current hospitalization, she suffered a mild/moderate COVID-19 infection with mild involvement of the respiratory system. The patient was hospitalized for COVID-19 in the Infectious Diseases clinic for fever (up to 38 °C) and non-producing cough, with a peripheral oxygen saturation of 90% which required oxygen administration using face mask with a fractional inspired oxygen concentration (FiO_2_) of 0.4.

Given the clinical picture dominated by cognitive symptoms, in the context of post COVID-19 infection, the suspicion of encephalitis was raised.

Upon admission, the laboratory tests revealed lymphocytopenia with lymphocyte count: 0.68 (1.2–3.4) × 109/L, blood sugar level: 320 mg/dL, interleukin-6 (IL-6) level: 28 ng/mL, C-reactive protein (CRP): 4.31 mg/dL, fibrinogen: 407 mg/dL, procalcitonin below 0.5 µg/L, D-dimer: 2.46 mg/L, total cholesterol: 260 mg%, ALT: 19 UI/L, AST: 20 UI/L, blood urea nitrogen: 59 mg%, creatinine: 0.66 mg/dL.

The chest computed tomography (CT) scan revealed subpleural ground glass opacities and infiltrates accounting for 30% of the total pulmonary surface. She was treated with Remdesivir 200 mg in the first day followed by 100 mg per day in the next four days, dexamethasone 8 mg/day for 7 days, paracetamol when she presented fever, enoxaparin sodium 2 × 0.6 mL, vitamin D 2000 UI/day, slow and fast insulin depending on the evolution of blood glucose level, hypotensive treatment (bisoprolol and candesartan cilexetil). The evolution was favorable with gradual improvement and fever regression after the first 4 days. Oxygen therapy was discontinued on day 9, and she was discharged after 2 weeks of being hemodynamically and respiratorily stable.

Oropharyngeal and nasopharyngeal swab test reverse transcription polymerase chain reaction (RT-PCR) at admission in Neurology clinic was repeated and was negative for SARS-CoV-2, she was afebrile and her respiratory and hemodynamic state was normal. Blood analysis revealed normal blood cell counts, and normal levels of CRP, fibrinogen, and ferritin. The arterial blood pressure at admission was 142/102 mm Hg, with a peripheral pulse of 92 beats per minute. The total cholesterol level was 230 mg% and the glycemic curve showed the following values: 170-212-153-273 mg/dL.

A large panel for neurotropic viruses was performed which included herpes simplex viruses 1 and 2, varicella zoster virus, cytomegalovirus, Epstein–Barr virus, HIV 1 and 2, hepatitis, all within normal values. For sample collection, 10 mL of venous blood was drawn in a biochemistry vacutainer Clot Activator (red lid). The sample was centrifuged at 3000 revolutions per minute (rpm) for 10 min and the serum was aliquoted in 2 mL Eppendorf conical tubes and used separately for each test.

The technique used was Enzyme-Linked Immunosorbent Assay (ELISA), a technique used in immunology to detect the presence of proteins (IgM or IgG for Herpes Simplex Virus, Cytomegalovirus, Epstein–Barr virus and Hepatitis Viruses). The antibodies are used to detect the specific proteins immobilized on the surface of microplate wells. For human immunodeficiency virus (HIV), the Antigen/Antibody Combo assay was used, a chemiluminescent microparticle immunoassay (CMIA) for the simultaneous qualitative detection of HIV p24 antigen and antibodies to HIV type 1 (HIV-1 group M and group O) and/or type 2 (HIV-2) in human serum.

The level of potassium channel complex antibody was 214 pmol/L. The antibodies against the voltage-gated potassium channel (VGKC)–complex were detected using the radioimmunoassay technique in serum samples.

A prior dilution of 1:10 using assay buffer (15 µL serum and 135 µL assay buffer) was performed. Duplicate aliquots of patient serum and control serum (one negative and two positive) were incubated with detergent solubilized VGKCs extracted from rabbit brain tissue and complexed with iod (I^125^) labelled α-dendrotoxin. After the first incubation, antigen–antibodies complexes formed and were immunoprecipitated by the addition of anti-human IgG. After a second incubation, the assay buffer was added, and the samples were centrifuged. Unbound I^125^-labelled α-dendrotoxin-VGKC complexes were removed by aspiration of the supernatant. The level of radioactivity remaining in the tube was proportional to the antibody level in the test sample. The lower detection limit at 2 standard deviations was 4.5 pmol/L and the positive assay cut off was ≥85 pmol/L.

Serology for *Borrelia* antibodies IgM and IgG were also negative. Antiphospholipid, anti-polynuclear, and antinuclear antibodies were negative.

After admission to the neurology ward, the patient initially presented with a regression of aphasia and truncal ataxia but with increasing temporal and spatial disorientation, a state of confusion and visual hallucinations that began to gradually improve under intravenous steroid treatment. After 6 days of treatment, the patient became more oriented and able to communicate better and answer simple questions.

The autoimmune panel for encephalitis and onco- and anti-neural antibodies (NMDA, AMPA, GABA-A, GABA-B, anti-Yo, anti-Hu, anti-Ri, anti-Ma2, anti-CV22, amphiphysin) were absent in the serum. Positivity for VGKC in the serum was revealed. A search for neoplasia was performed including a thoraco-abdominal-pelvic CT scan which was negative. Toxic, metabolic, hypoxic and acute infections derangements were excluded by laboratory tests. The patient and the family refused the lumbar puncture (LP) and we could not perform CSF testing.

**Figure 2:**
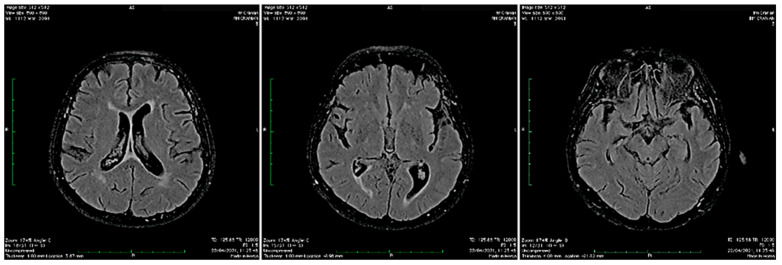
Cerebral MRI, FLAIR sequences revealing mild periventricular microvascular changes and cerebral atrophy.

**Figure 3:**
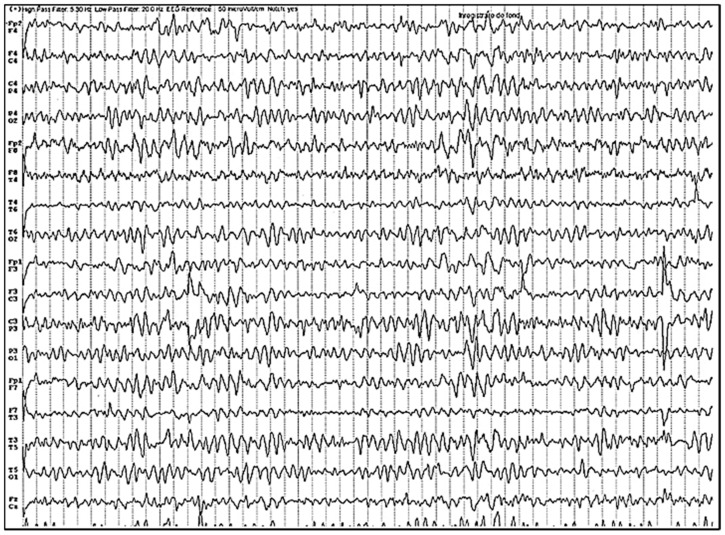
EEG examination revealing bilateral, slow-wave discharges, and spikes on the frontal, parietal and temporal derivations.

The patient then underwent a brain magnetic resonance imaging (MRI) including gadolinium contrast administration, which revealed only diffuse atrophy with no contrast enhancement (Figure 2). An electroencephalogram (EEG) was performed and revealed bilaterally, slow-wave discharges, and spikes on the frontal, parietal and temporal derivations sometimes interrupted by short episodes of fast frequencies with short amplitude (Figure 3).

The diagnosis was based on her neurological clinical presentation, the presence of psychiatric symptoms suggesting the involvement of the limbic system, the EEG abnormalities and the detection of specific antibodies recognized to be associated with limbic encephalitis. The diagnosis of autoimmune encephalitis was made according to the Graus criteria [18].

She was started on intravenous dexamethasone 2 × 8 mg per day with progressive improvement of the symptomatology during the first 10 days and then tapered using oral corticosteroids: 64 mg oral methylprednisolone for 14 days with progressive tapering of steroids with 4 mg at 2 days. Mycophenolate mofetil film-coated tablets 2 × 500 mg were associated with her treatment after the first 10 days. In the following days after receiving corticosteroids and antipsychotics, her medical condition improved and she was soon mobilized and able to walk.

She was discharged without major cognitive impairment and free of hallucinations on day 19 after admission. At follow up 3 months later, she had fully recovered with normal values of VGKC antibodies on immunosuppressive treatment.

#### 3.1.1. Literature Review

The search strategy identified twenty-one patients after removing the duplicates reports. The characteristics of included patients are represented in Table 1. A total of 105 publications were reviewed in full text after a first screening and a total of 20 articles (21 cases) that accomplished the inclusion criteria were finally involved in our review. In Table 1 we included details regarding the demographic data, the RT-PCR swab test results, the presence or absence of general and respiratory symptoms, the time interval between viral infection and onset of neurological symptoms and the main neurological manifestations. Other important characteristics are the electroencephalography (EEG) results, brain imaging, the CSF analysis (when obtained), the CSF RT-PCR for SARS-CoV-2, the evidenced autoantibodies, type of immunomodulatory treatment received and the outcome of the patients after treatment. 

The number of affected males was higher compared with women among the identified patients that were affected by autoimmune encephalitis 12/21 (57.14%). The age of the patients included in our study varied between 1–80 years. General and respiratory symptoms were mild or even absent in eight cases and the other three cases were admitted to the intensive care unit (ICU) due to respiratory failure or other complications. The most frequent EEG patterns presented generalized or focal slowing and epileptic discharges. In three cases, it was normal, and in the other four cases, it was not available. In ten cases, the brain CT/MRI was normal, and in other cases, brain hypodensity was observed on the CT scan (two cases) and hyperintense lesions edema with different localizations was observed on the MRI (nine cases).

Most reported patients developed autoimmune encephalitis within a few days to a maximum of 1 month after the onset of SARS-CoV-2 infection. In five cases the onset was concomitant, and there were four cases in which the RT-PCR swab test was positive after the onset of neurological symptoms (maximum 17 days).

Most of the cases included in our review presented anti-N-Methyl-D-Aspartate Receptor (NMDAR) antibodies (nine cases—42.85%), followed by anti-Myelin oligodendrocyte glycoprotein (MOG) antibody encephalitis (four cases—19.04%), two cases with anti-amphiphysin antibodies (9.5%) and just one case with contactin-associated protein (Caspr2) antibodies. In three cases (14.28%), the RT-PCR for SARS-CoV-2 from the CSF was positive (two patients with anti-NMDAR and one patient with anti-amphiphysin antibodies).

The immunomodulatory treatment administered consisted mainly of corticosteroids (sixteen cases), therapeutic plasma exchange (three cases), intravenous immunoglobulins (twelve cases), rituximab (one case) or different therapeutic combinations.

The majority of patients had a good evolution on immunomodulatory therapy, and no death was reported between the diagnosed cases.

#### 3.1.2. Long COVID and Neuro-COVID

On 5 May 2020, an infectious disease professor published a case in BMJ Opinion with persistent symptoms 7 weeks after SARS-CoV-2 infection, which drew attention to the idea of “long COVID” [19]. The growing number of articles published since then highlighted that post-COVID symptoms may persist or new symptoms may appear lasting many months after acute infection [1]. At present, various nomenclatures and time ranges (3–12 weeks) are used to define the syndrome, and a poor understanding of its etiology and potential treatment remains [2].

Tenforde et al. defined “long COVID” or “post-COVID syndrome” by collecting demographic and subjective symptoms data 14–21 days after a positive RT-PCR for SARS-CoV-2. They observed that acute infection may be followed by prolonged illness not only in severe cases but also in milder outpatient illness [38].

Brain hypometabolism demonstrated through positron emission tomography—computed tomography (PET-CT) in long COVID patients was associated with persistent functional complaints such as brainstem and cerebellar symptoms, cognitive impairment, insomnia, and hyposmia/anosmia [39,40]. The proven existence of autoantibodies in association with reduced brain metabolism suggest that long COVID syndrome could have an autoimmune mechanism.

The “post-COVID” condition was proposed by the (world health organization) WHO document released on 6 October 2021 to describe patients who had probable or confirmed SARS-CoV-2 infection with persistent symptoms at 3 months from the onset of COVID-19 and that can last at least 2 months without being explained by other alternative diagnoses [41]. Other authors such as Lledó et al. completed the definition and considered that the symptoms can be permanent, relapsing/remitting, or progressively improving over the course. These situations can determine hospital re-admission up to 27% after 6 months [2,42].

Acute neurologic complications or long-term sequelae of COVID-19 have been called “neuro-COVID” [43,44]. Patients admitted to the hospital with COVID-19 experience multiple neurologic complications, including cognitive complaints, seizures, encephalopathy, hypoxic brain injuries, altered mental status, psychiatric symptoms, cerebrovascular events, autoimmune encephalitis, and debilitating symptoms described as “brain frog” [1]. Numerous case reports, cohort studies and systematic literature reviews have reported persistent clinical neurological manifestations with headache, disturbances in smell and taste, and cognitive impairment which significantly impact the patient’s quality of life [2]. In clinical practice we also have encountered various acute and chronic neurological complications following the infection [2,5,6,39]. The most functional neurological complaints after apparent recovery from COVID-19 are memory disorders, anxiety, insomnia and general asthenia [14,17,39].

Many theories are used in an attempt to explain the persistence of symptoms although the underlaying cause has not yet been established. These symptoms include viral persistence [45] and inflammation in human olfactory epithelium such as in COVID-19-related anosmia [46], inflammatory damage secondary to the acute infection, aberrant immune responses [2] and mast cell activation syndrome [47].

Different studies with different designs have reported varied prevalence of neuro-COVID ranging from 4.1% [48] up to 84% in COVID-19 patients suffering from acute respiratory distress syndrome (ARDS) [49]. Fatigue is reported in up to 60% of patients following SARS-CoV-2 recovery until 12 months [50]. One possible mechanism explained in a narrative review link these symptoms with the congestion of the glymphatic system in the CNS with a toxic build-up due to reduced CSF drainage through the cribriform plate because of an increased resistance resulting from olfactory neuron damage and local inflammation [51].

Furthermore, postural orthostatic hypotension and tachycardia can be the result of chronic inflammation in the brainstem centers with dysfunction of the autonomic nervous system [1]. The glial cells are activated by the long-term immune response with chronic damage of the neurons [1].

A recent meta-analysis that included seventy patients with neurological manifestations and COVID-19 reported that 24.7% (almost one fourth) had GBS or its variants and others myelitis and/or encephalitis, diseases based on an autoimmune mechanism [52,53].

In the beginning of the pandemic, the morpho pathological and histopathological necropsy studies were limited by the restrictive rules. As the infection globally progressed, histopathological assessment for both CNS and PNS involvement started to be performed in specialized centers with trained pathologists, under strict safety regulations.

Presumably, a large number of cases with neurological involvement failed to be diagnosed [53]. Ischemic stroke was explained by direct vascular endothelial damage or as a result of hypercoagulability (with increased D-dimer level, prolongation of the prothrombin time, disseminated intravascular coagulation) that affects the cardiovascular system especially in the context of vascular comorbidities or in the presence of cardiovascular risk factors [53,54]. The brain autopsy has the potential role to make the differential diagnosis between the direct virus damage and the CNS and secondary vascular injuries in the context of COVID-19. The limited neuropathological data demonstrated the activation of the microglial cells, with perivascular T lymphocyte infiltration, CNS demyelination, such as acute myelitis and encephalitis/meningitis or PNS involvement [53].

The brain involvement is the result of hypoxic/ischemic encephalopathy with decreased oxygen supply in combination with neuroinflammatory response, in the context of systemic metabolic dysregulation as revealed by histological patterns [53,55].

Although viral encephalitis was expected to be a common complication, histopathological data confirm that encephalitis/encephalomyelitis in COVID-19 is the result of acute inflammation causing neuronal damage, vascular and demyelinating lesions rather than direct viral invasion [53,55]. Demyelinating lesions are caused by autoimmune reactions in the context of widespread inflammation and cases of multiple sclerosis onset concomitant with SARS-CoV-2 infection were reported earlier in the pandemic [53,56].

All these effects of the long COVID persistent mechanism can cause persistence of neurological symptoms and cognitive impairment. In addition, the mental health of people with COVID-19 was negatively impacted because of the fight with the disease itself and social distancing, isolation, or quarantine, which increase feelings of anxiety and loneliness, as well as sleep disturbances [1,57].

#### 3.1.3. Pathophysiology of COVID-19 Neurologic Complications

**General considerations**

COVID-19, especially severe forms, is associated with an aberrant inflammatory response with exuberant innate immunity activation, and hyperinflammation as well as increased levels of pro inflammatory mediators, cytokines and chemokines, CRP, ferritin, and D-dimers in parallel with lymphocytopenia, decreased CD4^+^, CD8^+^ T and natural killer (NK) and B cells [58,59,60,61]. The innate infected immune cells (mainly neutrophils and macrophages) by IL-6 upregulation promote the viral spread and lymphocyte apoptosis leaving un-infected host cells unprotected and reducing viral clearance [58,59,62]. Lymphocytopenia is negatively correlated with increased serum levels of tumor necrosis factor alpha (TNF-α), IL-6 and IL-10 and postmortem examinations of COVID-19 patients revealed lymphocyte depletion and spleen necrosis in parallel with lymphocyte death [58,62,63]. Furthermore, SARS-CoV-2 infection can result in the burn out of innate immune cells [64].

SARS-CoV-2 activates the immune antiviral response in acute infection but may also trigger an excessive systemic response with cytokine release accompanied by lymphocyte dysfunction, and lymphopenia, monocyte, and granulocyte abnormalities [65,66,67,68]. In the case of a poorly regulated immune response, the viral infection can progress to SARS and systemic inflammatory response syndrome (SIRS) with high mortality rates [7,65].

**The neurological involvement**

**Figure 4:**
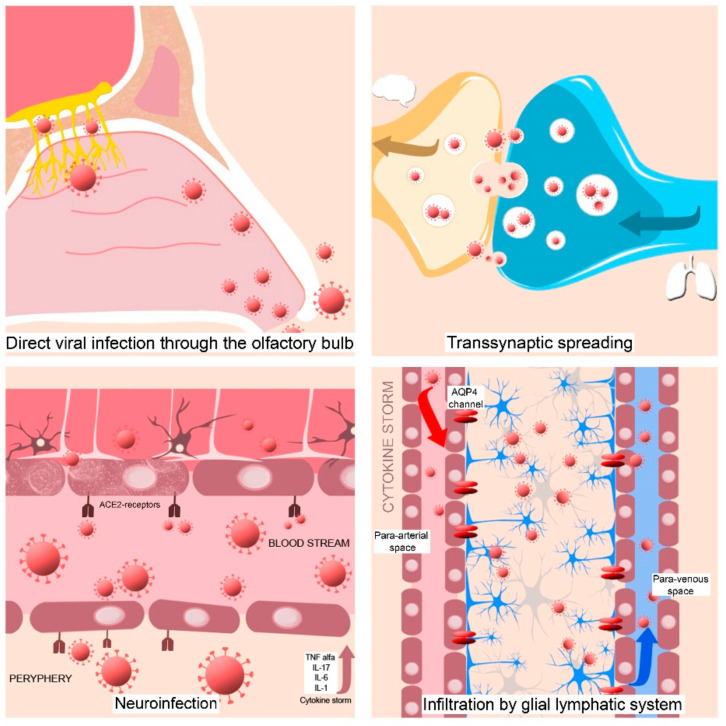
Graphical representation of the proposed mechanisms for neuro-invasion. Endothelial angiotensin-converting enzyme 2 receptors (ACE2-receptors), anti-aquaporin 4 (AQP4).

The main mechanisms discussed to explain neurological involvement are direct viral invasion, the systemic inflammatory response, and a prothrombotic state. The proposed mechanisms for neuro invasion are the following: (1) direct viral infection to the CNS through the olfactory bulb, (2) transsynaptic transmission, (3) neuro infection through the intermedium of blood circulation, and (4) the nervous distribution of the infected immune cells [58,69,70] (Figure 4).

1. The virus could enter via the olfactory nerve and move, across the cribriform plate to the brainstem by retrograde transport and then disseminate in CNS tissue [8]. This theory can be sustained by persistent anosmia/and or dysgeusia that is present in some COVID-19 patients [58,69,70] suggesting a nasopharyngeal route for CNS infection [4]. The inflammation of the olfactory bulb and olfactory mucosa caused by SARS-CoV-2 infection can explain the anosmia and the presence of CSF in the subarachnoid space of the meninges adjacent to the olfactory bulb can perpetuate the viral spreading in the CNS [53].
2. Another theory in the transsynaptic spreading of SARS-CoV-2 is from the peripheral receptors located in the lungs (chemoreceptors and mechanoreceptors) to the medullary cardiorespiratory center [9,71,72].
3. The virus could be distributed through systemic circulation and then in the cerebral blood flow. As a result of its interaction with the endothelial angiotensin-converting enzyme 2 (ACE2) receptors, it could penetrate the affected blood–brain barrier (BBB), which was compromised previously by the hyperactive immune responses [69].
4. The infected immune cells, such as leucocytes can infiltrate into the brain tissues through the glial lymphatic system, a lymphatic-like pathway in the brain initiated by peripheral inflammation [69,73,74] in the context of a cytokine storm.

The direct neuro invasion of SARS-CoV-2 was rarely detected [69] after lumbar puncture with CSF analyses, whereas other corona viruses, such as SARS-CoV and (Middle East Respiratory Syndrome Coronavirus (MERS-CoV) ribonucleic acid (RNA), were detected in the brain of the infected patients [75,76,77]. In the case of our patient the infection was cured at the moment of neurological diagnosis and the oropharyngeal swab RT-PCR test at admission was negative. However, isolated case reports outline when the virus has been detected in both CSF and brain tissue [8,58,78]. For example, an autopsy report evidenced the presence of SARS-CoV-2 in the brain tissue and capillary endothelial cells of the frontal lobe of a deceased patient due to COVID-19, confirming that neuro invasion may contribute to the neurological manifestations of this disease [69]. Even the detection of rare cases of patients in whom the test for SARS-CoV-2 was positive in the CSF still claims the assumption of direct neural-viral invasion. In critically ill COVID-19 patients, this evidence is suggested by the increased level of serum neurofilament light chain (sNfL) [79] which is a clue of direct neuronal injury. Multiple subcortical hemorrhages have been evidenced because of COVID-19 and an astrogliosis marker, the glial fibrillary acidic protein (GFAP) was found to be increased in plasma of moderate and severe COVID-19 patients and patients infected with SARS-CoV-2 suffering from disseminated encephalomyelitis [56].

The neuro-invasive potential of SARS-CoV-2 is assumed to be due to its affinity to entry in human cells through receptors of ACE2. Emerging data from studies have shown that viral S protein binds primarily to the ACE-2 receptors located on the surface of the respiratory epithelium, thereby mediating virus entry into the cells, followed by viral replication [65,80]. ACE2 receptors are also expressed in many organs, including the lungs, kidney, heart and brain, as neurons and, endothelial and glial cells (microglia, astrocytes, oligodendroglia) that can mediate the interaction between these cells and SARS-CoV-2 [8,58,81,82]. The neurological manifestations of COVID-19 may be a combination endotheliitis similar to that seen in the peripheral circulation [8,58], hyperactive immune responses and the supposed direct viral-neuro invasion [69].

The invasion of SARS-CoV-2 into olfactory support cells and perivascular cells rich in ACE2 receptors, the local inflammatory response, and the altered homeostasis for water and electrolytes decrease the function of the sensory neurons producing olfactory disturbances [83]. Another area enriched in ACE2 receptors is the mucous membrane of the tongue and oral cavity, which explains the ability of SARS-CoV-2 to increase the gustatory threshold by binding to sialic acid receptors [84].

The defensive system of the brain consists of microglia and astrocytes together with the immune cells. Microglia belongs to the innate immune cells with residency in the CNS having phagocytic properties against invading agents and possessing releasing proprieties of pro- and anti-inflammatory cytokines. In the area of circumventricular organs, (CVO) the BBB protection is reduced and makes this area more vulnerable to invasive agents (presumably also SARS-CoV-2) and peripheric neurotoxic molecules [64]. The local role of microglia in CVOs becomes even more important [64,85,86]. Astrocytes are neuronal cells of ectodermic origin, whereas microglia belong to the innate immune system and are of mesodermal origin, and together these cells contribute to the homeostasis of the CNS including the glymphatic clearance [64,87]. The astrocytes contribute to the formation of the BBB, which protects the brain from toxic and infectious invasions and all kind of peripheral influences [64,88] and regulate BBB permeability [64]. Microglia are known to have a role in brain maturation, synapse formation, and regulation of neurogenesis and gliogenesis. They are a source of trophic factors and pro- and anti-inflammatory cytokines and are recognized for the phagocytic activity and the contribution in myelination and remyelination processes [89,90] by regulating the activity of oligodendrocyte [64].

The pathophysiological mechanism of the CNS involvement could be also the result of a systemic hyperinflammation state with a “cytokine storm” and increased level of proinflammatory cytokines (TNF-α, IL-1, IL-6, and IL-17) in the peripheral circulation. These may impair neurovascular endothelial function and disrupt the BBB, leading to increased permeability [58,91,92,93,94] and facilitating interferences between peripheral adaptative immunity and brain innate immunity [58,91,92,94]. This process is followed by the activation of immune cells in the brain with a neuroinflammatory cascade that can induce CNS complications and even para infectious-autoimmunity [9,58]. The disruption of the BBB is exacerbated by different comorbidities, such as metabolic syndromes, increased age, and vascular conditions which allow different pathogens into the CNS, including viral particles followed by increased astroglial reactivity with astroglial death and further damaging of the BBB [95,96]. BBB can be crossed by a direct cellular transport through the vascular endothelium or by a “Trojan horse mechanism” that involves viral transport by the infected leucocytes that cross the BBB [52,53].

The brain’s defensive system and systemic inflammation can influence the CNS through both innate and adaptive immune systems through their cellular elements [64,97]. T- and B-lymphocytes can enter through the choroid plexus and disseminate in the perivascular space even when the BBB is intact aiming at invasive pathogens [64,98,99]. The damage of the BBB and dysregulations at this level results in increased pathological permeability so that leucocytes from the peripheral blood and blood-derived substances can infiltrate the brain parenchyma [1]. During the cytokine storm that accompanies severe COVID-19 infections, the BBB is compromised and is more permeable for immune cells and for circulating chemokines and interleukins that enter the CNS where they meet the local defensive systems represented by microglia and astroglia [64]. SARS-CoV-2 can invade these cells using the spike-like proteins (ACE-2 receptors) expressed on their surface [64]. Once inside the CNS, the virus binds to various cell types (neurons, astrocytes, microglia, and oligodendrocytes) via the ACE2 protein [100]. Penetration in the CNS is followed by the release of inflammatory mediators, which activate T-lymphocytes. The severity of neurological damage is influenced by the innate and adaptive immunity of the host [100].

Both microglia and astrocytes play a role in compartmentalized immune responses of the CNS followed by the autoantigen’s presentations, the activation of T-cells and B-lymphocytes with autoantibody production in conditions of BBB disruption [64,101]. Microglia and astrocytes can mediate the changes that take part in the brain as response to systemic inflammation and BBB breakdown with their’ failure to protect the entrance of peripheral immune cells in the brain [64]. Microglia have an amoeboid morphology, and the migration of the infected microglia from CVOs towards other brain regions may contribute to neuroinfection and neuroinflammation spreading [64]. When the resources of microglia are consumed because of immune involvement, their contribution to myelination, synaptic plasticity, the release of trophic factors, functional interactions with neurons, and regulation of neuronal activity may be compromised [64,102]. The area and surveillance of CVOs are affected if microglia turn dystrophic because of exhaustion [64]. Studies performed after herpes virus infection evidenced the cells belonging to oligodendrocyte lineage can be infected but also promote and sustain neuroinflammation when surviving [64].

In this way, the glial cells cooperate to maintain brain health and orchestrate the brain response to general and local aggression [64,103,104].

The composition of CSF is strictly controlled and represented by an ultrafiltrate of serum with a small number of leukocytes with different types compared with the blood with a reduced number of granulocytes and B-cells—the most abundant being CD4^+^ cells [105,106]. The CSF changes can give us diagnostic clues in some diseases that may affect the CNS, such as infections or hemorrhages.

SARS-CoV-2 RNA was rarely detected in the CSF of neuro-COVID patients [107,108]. Matschke et al. (2020) reported that they detected SARS-CoV-2 in up to 53% of brain autopsy specimens and it was more abundant in the lower cranial nerves [109]. In addition, their study proved a process of microglial activation and CD8^+^ T cells infiltration in the brain and the meninges of 79% of the analyzed patients [109].

The absence of pleocytosis in the presence of moderately elevated blood protein levels can be the result of T cells and B-cell lymphocytopenia due to lymphocytic apoptosis induced by viral replication [58,110,111,112] and does not exclude the possibility of an inflammatory process [58]. Negative-RT-PCR assay for SARS-CoV-2 in CSF samples may suggest that neurological complications are not the result of the virus entering the CNS but that a reduced CSF-viral load could also lead to false negative tests [58].

COVID-19 is accompanied by a complex immune dysregulation in the blood with a clonal expansion of CD8^+^ cells [113]. Heming et al. also identified changes in the CSF of neuro-COVID patients with exhausted or dysfunctional T cells, a relative lack of antiviral IFN producing CD4^+^ T cells, and an increase in a subset of monocytes with an antigen-presenting phenotype. This is a type of ineffective response that is different compared with other viral expansions of the CNS suggesting a potentially impaired antiviral reaction with a decreased IFN response in the CNS compartment of neuro-COVID patients [44]. Song et al. observed a compartmentalized immune response in the CNS with an expansion of B cells in the CSF and identified anti-SARS-CoV-2 antibodies in the CSF even though SARS-CoV-2 RNA has not been detected [44]. Even more, these authors evidenced a cross-reactivity against anti-glia and anti-neural epitopes, supporting the hypothesis that the immune-mediated mechanisms contribute to neurological complications of COVID-19 patients [44]. Autoimmune damage is thought to be the result of an aberrant immune response with non-recognition of self-antigens leading to an attack against host tissues [100].

**Autoimmunity**

Autoimmune diseases occur in genetic susceptible individuals as an aberrant immune response in recognizing self-antigens compared with non-self-antigens [114]. Viral infections have been considered to trigger autoimmune phenomena together with the genetic predisposition, age and surrounding environment [114].

In addition to multi-organ damage and complications, autoimmune-like lesions have been reported that suggest the involvement of the immune system in the pathology [115]. The immune system is a highly specialized entity whose role is to recognize and eliminate foreign particles, such as infectious agents or tumor cells [115]. The effector cells of adaptive immunity are T cells and B-lymphocytes which express on the surface-specialized receptors in recognizing specific regions of antigens called epitopes [115,116]. B cells are specialized in recognizing free antigens, whereas T cells recognize antigen peptide fragments presented by other cells (macrophages, and dendritic cells) that belong to a major histocompatibility complex (MHC). An important feature of adaptive immunity is the recognition and ability to react to foreign structures (e.g., SARS-CoV-2) but not to self-antigens [115,117,118]. Tolerance towards self-antigens is an essential factor to prevent immune response to self-structures and although self-reactive immune cells exist in circulation, these are usually suppressed [115,119,120,121]. However, the dysregulation of immune tolerance mechanisms in the context of inflammatory signals may lead to a loss of suppression and activation of reactive immune cells, resulting in the occurrence of autoimmune diseases, such as multiple sclerosis (MS), systemic lupus erythematosus (SLE) [115,122,123,124].

Several autoimmune diseases developed after SARS-CoV-2 infection such as GBS [125,126,127], autoimmune thrombocytopenia [128], SLE [11], MS [129,130], as well as autoinflammatory conditions, such as pediatric inflammatory multisystem syndrome (PIMS) and Kawasaki disease in children [131,132]. Some suggestive symptoms for systemic autoimmune diseases (e.g., extreme fatigue, myalgia, and joint pain) are present during acute infections and persist after viral resolution [133]. Other autoimmune phenomena consist of the formation of immune circulating complexes, its deposition with complement consumption, or the development of anti-DNA and antinuclear antibodies [9,134].

If we are to think from a historical perspective, “the pandemic flu” that existed one century ago was followed by an increased number of encephalitis cases [135], and other viral infections, such as the hepatitis C virus, were associated with an increase in the case reports of rheumatoid arthritis (RA) [115,136]. The occurrence of autoimmune encephalitis (e.g., anti-NMDAR encephalitis) is described after viral infections, particularly after herpes simplex but the role of viral infections in the pathophysiological mechanism of limbic encephalitis remains controversial [137]. Other infectious viruses have been also associated with autoimmune diseases such as cytomegalovirus infection with immune thrombocytopenic purpura and SLE, Epstein–Barr virus infection with SLE, and rheumatoid arthritis [115,138,139,140]. Other coronaviruses have been linked to autoimmune diseases such as multiple sclerosis (HCoV-229 E and HCoV-OC43) with antibodies against these viruses found intrathecally [141,142,143], suggesting a potential role in disease etiology [115,143,144]. SARS-CoV-1 infection was correlated with thrombocytopenia through a mechanism considered to be autoimmune in some previously infected patients [145,146]. The temporal association between these infections with autoimmune diseases as well as the temporal association between the cases reported after the SARS-CoV-2 infection may suggest a role of a causative trigger of COVID-19 in the development of CNS autoimmunity.

Reports of encephalitis accompanying acute SARS-CoV-2 infection, including presumed autoimmune encephalitis, are already widely published in the medical literature, but cases in which autoantibodies have been identified are rarer and are mostly isolated case reports or case series providing limited information about this disease [14,147].

Most reported cases of postinfectious encephalitis accompanied severe cases of COVID-19 and occurred relatively early after SARS-CoV-2 infection with an average of 14.5 days according to Siow et al. [13]. This differs from our case, as the infection had a mild evolution and the neurological symptoms set in insidiously and progressed, possibly over several weeks, with the neurological diagnosis of autoimmune encephalitis being made approximately 4 months post-infection. From the data reported by Siow et al., the average age of the patients was 59.4 years, with a similar proportion of males and females, and the most common comorbidities were hypertension (45.5%), diabetes mellitus (26%) (such as in our patient), and hyperlipidemia (24%) [13]. These comorbidities may suggest a proinflammatory status before SARS-CoV-2 infection—a favorable background for the occurrence of the cytokine storm [59].

**Figure 5:**
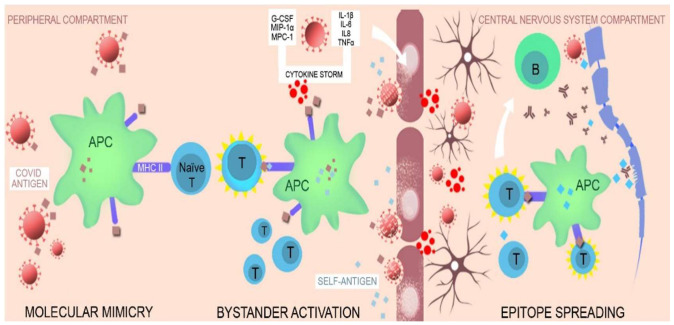
Graphical representation of the proposed mechanisms for viral induced autoimmunity. Key mechanism for viral induced autoimmunity: **(1) molecular mimicry**-cross-reactive response against virus and self-antigens leading to lymphocyte activation; **(2) bystander activation**—the released self-antigens are taken by the antigen presenting cells(APC) and presented to naïve T cells which become responsive. Simultaneously, the innate immune system triggers the production of proinflammatory cytokines and chemokines and initiate self-tissue destruction that generate self-tissue antigens mimicking viral antigens; **(3) epitope spreading**—the viral infection produce futher damage and release of more self-antigens with subsequent activation of auto-reative T cells. When the damage involves the central nervous system it triggers T and B cells activation and maintain a pro-inflammatory environment.

Triggering factors that contribute to this mosaic of autoimmunity are multiple and include environmental factors, immune defects, and genetic predisposition. The key mechanisms for viral-induced autoimmunity are:

- Molecular mimicry;
- Bystander activation [114,115];
- Epitope spreading [114] (Figure 5)

Molecular mimicry means that there is a structural similarity between a self-protein and foreign antigen and both are recognized by the same lymphocyte receptors, resulting in a crossed reactivity [115]. In the case of COVID-19 infection, it is described as shared immunological epitope between the virus and the human host, a structural similarity of the virus antigens with self-antigens which lead to T- and B-cell activation and a cross reactive response of the body against virus and self-antigens [114,148,149]. The human proteome shares multiple peptides with SARS-CoV-2 epitopes [68]. Some studies evidenced a similarity between sequencies of SARS-CoV-2 proteins and human proteins found in multiple organs/body tissues (neurological, vascular, and cardiac) which demonstrate the potential of immune crossed-reactivity in these regions to be recognized as epitopes by B- and T-cells [148,149,150].

Bystander activation is the result of self-antigens release from damaged tissues [114]. These antigens are taken up by the antigen presenting cells (APC) and presented to naïve T-cells (autoreactive T cells) which become responsive. Bystander activation is explained by the activation of immune cells as a result of self-antigens liberation by the action of the virus, and these self-antigens are not normally exposed to the immune system in the absence of an infectious trigger, for example [115]. The innate immune system, which is the first line of defense, reacts to SARS-CoV-2 infection by increasing the production of proinflammatory cytokines (IL-1β, IL-6, IL-8, and TNF-α) and chemokines (G-CSF, macrophage inflammatory protein 1α: MIP-1α, and monocyte chemoattractant protein 1: MPC-1), and “cytokine storm” initiates self-tissue damage that generates self-tissue antigens that can mimic COVID-19 antigens [100].

“Epitope spreading” means that the viral infection produces damages and release of more self-antigens with further activation of autoreactive T-cells that target new self-epitopes [114]. When this damage occurs in the CNS (axonal membrane, myelin sheets, and BBB) the resulting self-antigens are taken by antigen presenting cells (APCs) and presented to naïve T cells that become activated and further trigger the activation of B cells with autoantibody production and maintain self-tissue damage with a release of more CNS-specific self-antigens. These autoantibodies targeting CNS tissues may produce acute and long-term damage and can lead to autoimmune or neurodegenerative diseases (Parkinson’s disease, and Alzheimer’s disease) after COVID-19 [100,114].

Endogenous viral proteins are presented by the APC through the MHC class I pathway, whereas antigenic epitopes from exogenous viral proteins are presented through the MHC class II pathway to host T cells. Generally, class I molecules are not found on neurons [151].

In COVID-19, the chemotactic call of lymphocytes towards the lung tissue together with increased apoptosis in peripheral blood and bone marrow shut down leads to transient lymphopenia and immunosuppression with a loss of tolerance to some self-antigens [65]. This redistribution of immune cells also affects the innate components of immunity, such as dendritic cells, monocytes, and macrophages, that have the role of permanently scanning the tissues to identify non-self-structures against which an inflammatory response would be triggered while not reacting towards self-structures [152]. Damage to immune system components, immune system redistribution, and aberrant immune reconstitution during the recovery phase after viral resolution when the lymphocytes level increase again in the context of deviated immune reconstruction may lead to unregulated response with failure or poor recognition of some self-antigens with a triggering of autoimmunity phenomena in the context of immunodeficiency that affects both innate and acquired immunity [65]. Even thrombotic phenomena have been associated with the presence of antiphospholipid antibodies and may occur in the context of an antiphospholipid syndrome [153]. The risk of developing autoimmunity phenomena is probably related to genetic predisposition, especially the human leucocytes antigens (HLA) gene polymorphisms and other genes [154], the existence of autoimmune diseases in the family, and the preexistence of some autoantibodies such as antinuclear antibodies [65,155,156].

Due to the association between coronavirus infection and autoimmunity and the similarity of SARS-CoV-2 with these viruses, it is reasonable to assume that a link exists between SARS-CoV-2 infection and some later diagnosed autoimmune diseases [115]. Elevated levels of autoantibodies, such as antinuclear antigen (ANA) antibodies, anti-60-kDa and anti-52kDa SSA/Ro antibodies and antiphospholipid antibodies (aPLs), have been evidenced, especially in patients who have experienced severe forms of COVID-19 [115,157]. In addition to these antibodies known to be associated with autoimmune diseases, other antibodies such as autoantibodies against type I-IFNs or directed against other cytokines including GM-CSF, IL-6, and IL-10 have also been identified. In patients with neurological symptoms associated with severe COVID-19 cases, autoantibodies in serum and sometimes in CSF of neuronal targets have been identified (anti Yo antibodies, anti-NMDA receptor antibodies, and anti-myelin antibodies) suggesting a dysregulation of the immune system in SARS-CoV-2 infection, which can make a connection with GBS, MS, myelitis, and encephalitis cases reported after the infection [5,12,13,14,115].

These mechanisms may coexist and are not necessarily exclusive and could make the link between systemic inflammation and systemic diseases followed by neuroinflammation, autoimmunity, and neurodegenerative processes explaining the cognitive and neurological consequences of COVID-19 [44,64].

## 4. Discussion

The number of published articles with autoimmune neurological disorders and neuro-COVID or long COVID cases with neurological symptoms is continually growing. Consequently, this will likely lead to a better understanding of the epidemiology and pathophysiological mechanisms underlying these manifestations [1].

The role of SARS-Co-V-2 in producing direct CNS infection or triggering an autoimmune-mediated response is still under debate [4,8,9,10,158]. Many reports in the literature demonstrate the association between SARS-CoV-2 infection and acute neurological manifestations but little is known about the postinfectious and long-term effects, including possible neurological sequelae of the infection [14,58,70,147,159]. Neurological complications of COVID-19 may occur as a result of direct virus injury, para- or postinfectious immune-mediated or inflammatory reactions triggered by the SARS-CoV-2, or secondary to the systemic inflammatory response syndrome [71,109,115].

We described the case of a patient with autoimmune encephalitis with VGKC antibody with a possible connection with COVID-19, given the onset after about 1–2 months with slow insidious evolution after SARS-CoV-2. The evolution was favorable under corticosteroid treatment. Our patient’s prompt response to treatment supports early diagnosis and appropriate therapeutic intervention.

VGKC, a protein complex, is present on the membranes of the neurons in the CNS and PNS implicated in the repolarization of the post synaptic membrane. Antibodies directed against the VGKC have been demonstrated to be involved in several CNS and PNS autoimmune diseases and are characterized by a wide range of neurological complications including epilepsy, limbic encephalitis and acquired neuromyotonia [160,161].

VGKC-antibodies are targeted to different epitopes of the VGKC, most of them being directed to anti-leucine-rich glioma inactivated 1 (LGI 1) and Caspr2 [162,163]. These autoantibodies created by the immune system binds to these antigens from the neuronal surface and determine neuronal surface antibody-mediated autoimmune encephalitis [164]. The clinical relevance of these different immune responses is important, because antibodies against Caspr2 and LGI1 are associated with different well-defined clinical syndromes.

LGI1 is a neuronal secreted protein, and not a structural component of VGKC. The LGI1 together with a presynaptic protein ADAM 23 (a metalloprotease protein 23 and a disintegrine) and a postsynaptic protein ADAM22 (a metalloprotease protein 22) create a transsynaptic protein complex. This protein complex regulates α-amino-hydroxy-5-methyl-4-isoxazolepropionic acid receptor (AMPAR) and VGKC currents, is implicated in the synaptic transmission of the neuronal excitability, and plays an important role in the inhibiting signaling of neurotransmission in SNC [165,166]. The LGI 1- antibodies, act by interfering with this protein–protein interaction between LGI1 and ADAM 22/ADAM23 in the temporal cortex and hippocampus [167]. An anatomo-pathological study showed infiltration in the temporal lobe with T -helper cells (CD8+ cytotoxic type) with direct T-cell-mediated toxicity, complement activation, and complex immune activation in a LG1 encephalitis antibodies patient [168]. This finding demonstrates that all adaptive immune system components are activated in LGI1 antibodies encephalitis.

The LGI1 antibodies are presumed to increase neuronal excitability, which can manifest through epileptic seizures [167]. The majority of patients with anti-LGI1 encephalitis present clinical signs of limbic encephalitis: temporal lobe epileptic seizures, memory disturbances and behavioral changes. The seizures can be focal, generalized tonic-clonic or faciobrachial dystonic seizures [169,170,171]. Other characteristic symptoms that these patients can develop are sleep disorders (especially insomnia) and autonomic dysfunction [169,170]. The disease evolves progressively with a maximum severity at 3–6 months [162,169,172]; the data are similar to those reported in our patient.

The diagnosis of LGI1 encephalitis is based on LGI1 antibody detection in serum and CSF [170]. Brain MRI usually shows hypersignal intensities in the medial temporal lobes in the hippocampal region, but it can be normal in up to 10–25% of cases [169,170,173]. Other findings that were reported were hypersignal lesions in T1- and T2-weighted images located in the basal ganglia. Patients with LGI1 antibody encephalitis can develop mesial temporal sclerosis and hippocampal atrophy after remission of the acute phase [169,173].

The treatment of patients with LGI1 antibody encephalitis consists of methylprednisolone, IVIg and plasma exchange. Associated immunotherapy with azathioprine or mycophenolate mofetil with oral corticosteroid can be used as a therapeutic scheme for sustained improvement [171].

Caspr2 is a cell adhesion molecule from the neurexin IV family group. Caspr2 in conjunction with contactin-2 establishes a transmembrane axonal protein complex in the juxtaparanodes region of the myelinated axons. This Caspr2-contactin-2 complex is mostly expressed in the CNS inhibitory neurons and controls the axonal excitability, by avoiding repetitive firing and stabilizing conduction through the nodes of Ranvier [174,175,176]. Caspr2 interacts with contactin-2 in the presynaptic region and with gephyrin in the postsynaptic region [177].

Caspr2 antibodies affects the inhibitory interneurons in the hippocampal region, disturbing the interaction between Caspr2 and contactin-2 and altering gephyrin gathering in the postsynaptic region [178]. Anti Caspr2 antibodies encephalitis is a rare disease, usually occurring in men between 60 and 70 years of age. The common clinical signs are multifocal, diffuse encephalitis or limbic encephalitis [169,179]. The majority of patients have cognitive deficits, epileptic seizures and sleep disturbances with insomnia. Autonomic dysfunction with hyperhidrosis and cardiac autonomic dysfunction may be present. Other clinical features can be neuropathic pain and weight loss [169,180]. Because the Caspr2 protein is expressed in both CNS and PNS axons, these patients can develop a syndrome of associated peripheral nerve hyperexcitability, which presents with neuromyotonia, fasciculations and cramps, that can precede the encephalitic symptoms [169,180]. This clinical context with sings of PNS and CNS dysfunction is classified as Morvan syndrome [169,181]. Sometimes the tumor screening in these patients finds different types of tumors (thymoma, endometrial and lung cancer) [181].

Brain MRI can be normal, but increased signal activity in T2-weighted sequences in the medial temporal lobe, most often bilaterally, may be detected. CSF analysis can be normal or may show increased protein level and cell count in some patients [163,182]. The diagnostic confirmation is made by the detection of Caspr2 antibodies in VGKC- radio-immunoassay screen in serum and with additional confirmatory testing in CSF [163,182]. First-line treatment of autoimmune encephalitis includes glucocorticoids, plasmapheresis and IVIg, and should be started immediately after diagnosis. Rituximab and cyclophosphamide can be used as second-line treatments [163,182,183,184]. Most of the autoimmune encephalitis patients have a good outcome; although clinical relapses can occur in up to 25% of patients, they do not remain with symptoms or disability after disease [172].

The underlying neoplasms were ruled out in our patient, there was no evidence of acute infection, and autoimmune damage occurred after recovery from SARS-CoV-2 infection. In this context, our patient history, neurological presentation and the presence of autoantibodies with a clinical picture of CNS involvement suggests an autoimmune reaction that could be triggered by SARS-CoV-2 infection. The body’s response to SARS-CoV-2 infection appears to play a critical role both in the acute phase and in the subsequent clinical manifestations associated with long COVID [68]. It is assumed the neurovascular dysfunction and neuroinflammation [58,185] associated with the CNS aberrant innate immune signaling persistence induced by SARS-CoV-2 infection and neurological symptoms in COVID-19 may be caused by the direct invasion of the virus into the nervous system or by immune-mediated para infectious conditions [18,49,186,187,188].

Another alternative hypothesis would be a random association in the temporal sequence of autoimmune encephalitis with SARS-CoV-2 infection or the existence of a neoplasm with the appearance of a paraneoplastic syndrome with neurological manifestations, but the CT scans were negative and all onconeural testing was negative. Of course, it is necessary to monitor and scan the patient’s dynamics to rule out tumor occurrence.

The most common findings on MRI seen in patients with encephalitis accompanying SARS-CoV-2 infection include white matter changes with hyperintensities, and hemorrhagic lesions [189], but a few case reports of autoimmune encephalitis also report normal brain MRI imaging [13,16,17]. This may be the result of milder encephalitis or the brain changes did not develop until the brain imaging was performed [13]. Other findings in MRI performed in deceased patients with COVID-19 evidenced asymmetry of the olfactory bulb and changes in brain parenchyma supporting the trans-nasal virus spread and then further invasion of the brain [64,190]. Usually, EEG showed patterns of general slowing [191].

These temporal associations between COVID-19 and anti-VGKC antibodies and VGKC-mediated autoimmunity are important for the therapeutic decision (steroids, therapeutic plasma exchange, or intravenous immunoglobulins). As with our patient, immunotherapy seems to have had a beneficial effect in some cases [16,24]. The case reported by us and the cases of autoimmune encephalitis in which autoantibodies were detected suggest that autoimmune responses with a CNS involvement could be triggered by the infection with SARS-CoV-2 and growing data suggests and enhanced immune response in the pathophysiology of neurological disorders that occur after COVID-19 [3,4,17,39].

The purpose of this presentation and review of the literature is to develop a rapid diagnosis work up and to draw attention to this type of pathology in the presence of a suggestive clinical picture after toxic, metabolic, infectious or structural factors have been excluded with a proper treatment implementation.

The main limitations of our study are the relatively small patient group, the heterogeneity of the patient data and the incomplete workup in the reported cases. However, there are only a few cases reported in the medical literature of autoimmune encephalitis with proven autoantibodies as a possible complication of SARS-CoV-2 infection, despite the huge number of publications addressing COVID-19. Our literature review and the illustrative case reported by us may provide useful preliminary data that can be completed along with the understanding of the pathophysiological mechanism underlying this complication.

## 5. Conclusions

The identification of certain autoantibodies in some cases raises the question about the possibility of SARS-CoV-2-induced autoimmunity. Current clinical observations and laboratory data indicate a dysregulation of the immune system that follows SARS-CoV-2 infection that could be a starting point in triggering autoimmune diseases. It is worth analyzing different autoantibodies in different chronic manifestations following acute infection with SARS-CoV-2, including long COVID syndrome, because autoimmune mechanisms may at least partially explain the associated persistent phenotypic manifestations. In a clinical suggestive context, a systematic screening should be performed because this can lead to proper therapeutic decisions with functional implications in some patients.

Autoimmune encephalitis may be a consequence of COVID-19 and a causal relationship may be inferred from the temporal sequence of events. A diagnostic algorithm to prevent negative outcomes due to the evolutionary nature of the disease and disruption of the pathophysiological substrate and autoimmune attack are essential. In conclusion, only a few cases of autoimmune encephalitis following COVID-19 with autoantibodies have been described so far, and it is important to consider the possibility that the autoimmune process can be triggered by SARS-CoV-2 infection, especially if the autoimmune disease begins after a certain period when the respiratory symptoms have disappeared, and the infection is considered cured.

We think that this report and literature review will stimulate further investigations to discover specific antibodies in autoimmune encephalitis. The long-term implications and the impact of the new variants of COVID-19 are of increasing concern as the pandemic continues worldwide. In the future, the analysis of several studies and laboratory data of published cases will help a better understanding of the mechanisms that link SARS-CoV-2 infection with autoimmune diseases that may arise in susceptible individuals.

The study of this long COVID or post-COVID syndrome requires specialized units that bring together medical specialties forming a multidisciplinary team for a holistic approach. Indeed, the neurologist is an important element in this team given the multitude of long-term neurological complaints in patients who have experienced an acute infection.

## Figures and Tables

**Table 1:** Summary of the cases published in the literature.

Authors	Age (Years), Sex	RT-PCR Test Swab	General and Respiratory Symptoms	Days between Infection and Neurological Onset	Neurological Symptoms	EEG	Brain CT/MRI	CSF	CSF RT-PCR	Autoantibodies	Immunomodulatory Treatment	Outcome
Guilmot et al., 2020 [16]	80, M	P	Mild	NA	Seizures	Generalized slowing	Normal brain MRI	9 cells/mm^3^	N	CASPR2 positivity on CFS and blood	Corticosteroids, therapeutic plasma exchange	Seizure’s control
Guilmot et al., 2020 [16]	71, NA	P	Mild	5 after	Gait ataxia, akathisia, early delirium and choreiform involuntary movements of the upper limbs	Generalized slowing	Brain CT: normal	Normal	N	Serum anti-gangliosides antibodies (anti-GD1b IgG) (brainstem encephalitis)	NA	Good clinical evolution
Grimaldi et al., 2020 [17]	72, M	P	Fever 38.5 °C; Thoracic scan: peripheral bilateral ground glass lesions, opacities	17 after	Bilateral upper limb tremor, impossible walking, ataxia, dysarthria, upper limb dysmetria, diffuse myoclonus	Symmetric diffuse background slowing	Normal brain MRI	Normal cell count, proteins: 49 mg/dL, negative oligoclonal bands	N	Autoantibodies directed against the nuclei of Purkinje cells, striatal and hippocampal neurons evidenced by nerve tissue immunostaining	Corticosteroids, IVIg	Improvement of neurological condition, myoclonic seizures control
Ayuso et al., 2020 [20]	72, F	P	Mild	30 after	Slight inattention, disorientation, down beat nystagmus in all gaze positions, truncal ataxia, reflex myoclonus in the face and both arms	Normal	Brain MRI: hyperintense lesions in the caudal vermis and right flocculus with contrast enhancement in the floor of the fourth ventricle	10 leukocyte/mm^3^, glucose: 70 mg/dL, absence of oligoclonal bands	NA	Anti GD1a antibodies (Bickerstaff encephalitis)	1000 mg IV methylprednisolone daily (for 5 days)	A significant improvement
Monti et al., 2020 [21]	50, M	P	Absent	4 after	Confabulations and delirious ideas, then in evolution: focal motor seizures, impaired awareness, oro-facial dyskinesia, automatisms, refractory status epilepticus	General dezorganization,	Brain MRI: negative	76 cells, oligoclonal bands; negative for fungi, viruses, bacteria	N	NMDAR antibody present in CSF	Metilprednisolone, IVIg, therapeutic plasma exchange	4 months after onset: discharged in good condition, without neurological deficits
Panariello et al., 2020 [22]	23, M	P	Fever, desaturation (90% O_2_ saturation in inhaled air), three weeks later with dysautonomia (fluctuations in respiratory rate, blood pressure, cardiac rhythm, body temperature)	Same time	Anxiety, psychomotor agitation, auditory hallucinations, persecutory delusions, global insomnia; three weeks later: non-verbal, non-responsive to commands; able to move the extremities and react to noxious stimuli	Theta activity (6 Hz), unstable, non-reactive to visual stimuli and without asymmetries	CT cerebral scan was negative for neuroanatomical acute anomalies	Hematic appearance with 960 (red and white cells)/mm^3^; glucose: 70 mg/dL, proteins: 65.4 mg/dL, Ab anti NMDAR: positive	N	Positive Ab anti NMDAR in CSF	High doses of dexamethasone and IVIg	Clinical condition in amelioration at the date of publishing the article
Alvarez-Bravo et al., 2020 [23]	30, F	P	Fever at admission; later in evolution hypovolemic shock after a surgical intervention for teratoma; admitted to ICU	3 before	Paranoid ideation, psychomotor agitation, visual hallucinations, dysarthria; three days later: focal and generalized seizures; in evolution: decreased level of consciousness, generalized choreo-dystonic movements, blepharoclonus	Epileptic discharges in the left frontotemporal region; in evolution: delta brush pattern	A brain CT scan at admission was normal; brain MRI study performed a few days later: hyperintensities in the left hippocampus.	WBC: 44/mm^3^ (90% lymphocytes); proteins: 54.5 mg/dL	N	Anti NMDAR antibody positive in serum and CSF	Methylprednisolone, IVIg; later due to lack of appropriate response: rituximab	Discharged 70 days later with cognitive sequelae, memory disorders, emotional lability and sent to rehabilitation
Manganotti et al., 2021 [24]	37, M	P	Severe, ICU admission with respiratory support	Same time	Convulsive status epilepticus	Persistent generalized epileptic discharges	Brain CT scan: normal; Brain MRI scan: normal, negative contrast enhancement	1 mononuclear WBC, protein: 56.7 mg/dL, glucose: 66.1 mg/dL	N	Anti amphiphysin antibody	IVIg 0.4 mg/kg/5 days	Complete clinical recovery, free of seizures and EEG normalization, no respiratory support
Sarigecili et al., 2021 [25]	7, M	P	Absent	3 before	Behavioral and mood changes, encephalopathy, abnormal movements, seizures, autonomic instability, ataxia	Widespread delta waves	Brain MRI: normal	Normal except NMDAR antibodies	N	NMDAR antibody in CSF	IVIg, TPE, steroids (in this order)	Significant improvement to an ambulatory state
Allahyari et al., 2021 [26]	18, F	P	Shortness of breath, dry coughs	Same time	Mood changes, depression, anhedonia, lack of concentration, seizures	NA	Brain CT at the beginning: brain edema; brain MRI after 3 days: normal	13.900 WBC/mm^3^ (87% neutrophils)	P	NMDAR antibody in CSF	Corticosteroids, IVIg	Discharged with full recovery
Burr et al., 2021 [27]	2, F	P	Absent respiratory symptoms; general symptoms: fever, reduced oral intake, constipation	Same time	Poor sleep, fussiness, no talking, hyperkinetic movements of the arms, legs, head, seizures	NA	Brain MRI (native and with contrast): normal	7 leucocytes/mL (89% lymphocytes); glucose: 56 mg/dL; proteins: 25 mg/dL	N	NMDAR antibody positivity in the serum and CSF	5 days: intravenous methylprednisolone (30 mg/kg/day) followed by IVIg 2 mg/kg	Gradually resolution of the abnormal movements and encephalopathy; return to base line 2 weeks after discharge
Peters et al., 2021 [28]	23, M	P	Absent	14 after	Left sided headache and dysesthesias; after 5 weeks: personality changes, cognitive slowing, mild inattention, delayed recall, decreased verbal fluency	Epileptiform spikes and left posterior temporal rhythmic delta activity,	Brain MRI: normal; brain MRI repeated after 2 weeks: left hemispheric leptomeningeal enhancement; diffuse left hemispheric hyperintensity on FLAIR	1 WBC; proteins: 36 mg/dL; glucose: 78 mg/dL; Repeated LP after 2 weeks: 57 WBS/mm^3^ (50% lymphocytes)	N	Serum positivity for MOG IgG antibody and CSF negativity	Methylprednisolone 1 gr/day (5 days) and then oral steroid taper	Improvement of the cognitive symptoms, resolution of the headache; total resolution of the symptoms in 8 weeks.
Gaughan et al., 2021 [29]	16, F	P	Fever, sore throat, tachycardia	3 after	At admission: insomnia, anorexia, visual and auditory hallucinations, ritualistic behaviors, paranoia; mutism five days later with no voluntary activity; fecal and urinary incontinence; bilateral limb rigidity and tremor in evolution	An excess of delta and theta activity, more expressed in the right temporal derivations	Brain MRI: two tiny T2/FLAIR hyperintensities located in centrum semiovale bilaterally, without diffusion restriction and without contrast enhancement	WBC: 2 cells/ mm^3^; proteins: 43 mg/dL; glucose: 2.9 mmol/L	N	Anti-GAD antibody transiently positive in serum, negative in CSF	IVIg 0.4 mg/kg/day (5 days), followed by methylprednisolone 1 g per day, 3 consecutive days followed by a second course of IVIg	Discharged at home on day 98 after admission with significant cognitive and physical difficulties and sent to rehabilitation
Oosthuizen et al., 2021 [30]	52, M	D0: N D17: P	Tachypnea (20 bpm), fever (37.7 °C)	17 before	Progressive gait instability, multidirectional gaze-evoked nystagmus, truncal ataxia, dysatria	EEG at admission: normal	Brain CT at admission: central midbrain hypodensity Brain MRI: characteristic for brainstem encephalitis	CSF: pleocytosis (49 lymphocytes/ mm^3^, 2 PMN/ mm^3^); proteins: 37 mg/dL, glucose: 3.6 mmol/L	P	Onconeural antibodies positive for amphiphysin in serum	Prednisone (1 mg/kg/day)	Discharged on day 36, able to walk independently with a mild emotional lability
Vraka et al., 2021 [31]	1, F	P	Fever, hypertension	3 before	Altered consciousness, seizures, drowsy, hypotonic, swallow difficulties, in evolution decorticate posturing and GCS 5 points	EEG: diffuse slow wave background activity, no epileptiform discharges	Brain CT: biemispheric white matter hypodensities; Brain MRI: bilateral widespread white matter hypersignal abnormalities (including splenium corpus callosum, thalamus and pons)	WBC: 10/mm^3^	N	MOG antibody positive in serum	Steroid therapy	At discharge the patient was able to sit and walk a few steps, eat and drink normally but with cortical visual impairment with gradual improvement after four months
Ahsan et al., 2021 [32]	7, F	D0:N D9: P (Ab IgG)	Abdominal pain, fever	Same time	Status epilepticus, aphasia, encephalopathy, prolonged Todd’s paralysis; after o week: headache, dysarthria, altered mental status	EEG: cerebral slowing with left focal slowing	Brain MRI: cortical edema, peri Rolandic and posterior parietal lobe restricted diffusion	CSF at admission, WBC: 132/mm^3^ (64% lymphocytes), proteins: 54 mg/dL, glucose 73 mg/dL	N	MOG antibody positive in serum 1:40 at admission and 1:100 after 7 days	IVIg 2 g/kg over 3 days	She was discharged with improved condition, free of seizures with mild dysarthria
Valadez-Calderon et al., 2021 [33]	28, M	P	Mild symptoms of COVID-19	14 after	Somnolence, incoherent speech, auditory hallucinations, suicidal ideation, generalized tonic-clonic seizures, catatonic symptoms; two days later: status epilepticus	Subcortical dysfunction in frontal, temporal and occipital regions	Brain MRI: hyperintensities in the bilateral anterior cingulate cortex and temporal lobes	NA	N	NMDAR and GAD65/67 antibody positive in serum and CSF	Methylprednisolone 1 g/daily (5 days), followed by IVIg 0.4 g/kg/day (5 days)	Clinical improvement; at six weeks follow-up: still presents mood changes, irritability, agitation episodes
Durovic et al., 2021 [34]	22, M	P	Fever, general weakness	3 after	Severe headache, neck stiffness, a loss of smell and taste; days 16: mild impairment in executive functions	NA	Brain MRI: multiple disseminated T2 and FLAIR hyperintensities, no contrast enhancement	Cells: 31/mm^3^; proteins: 39.9 mg/dL, glucose: 64 mg/dL, lactate 11.9 mg/L	N	MOG (1:640) and mGluR1 (1:40) antibody positive in serum and CSF	Methylprednisolone 1 g/daily (5 days)	Two months later the patient presents no residual symptoms
McHattie et al., 2021 [35]	53, F	D0: N D14: P	Fever, myalgia; later in evolution: hypoxemia requiring oxygen therapy and transfer in ICU; day 17: dysautonomia (hypotension, bradycardia)	14 before	Palilalia at admission, after three days: confusion, urinary retention then severe echolalia, echopraxia, behavioral disinhibition; focal seizures, left side weakness	Slow activity, no epileptiform discharges	Brain CT with intravenous contrast at admission: normal Brain MRI: hyperintensity in the left amygdala, left anterior putamen, subtle changes in the left amygdala (FLAIR)	WBC: 141/mm^3^ (100% lymphocytes);	N	NMDAR antibody positive in CSF (1:100), negative in serum	IV and oral steroids, IVIg, Tocilizumab	One month later: remission of palilalia and seizures, improvement of cognitive functions, left side weakness
Sánchez-Morales et al., 2021 [36]	14, M	N	None	NA	Altered behavior and mental status, orolingual dyskinesias, insomnia, seizures	NA	Performed, result NA	Cells: 2/mm^3^, proteins 23 mg/dL	P	NMDAR antibody positive in CSF	Methylprednisolone, IVIg	A partial recovery of the neurologic symptoms with seizures control but with psychiatric symptoms persistence
McAlpine et al., 2021 [37]	30, M	P	Fever and malaise	Gradually, after a few days	Initially hypersomnia, then insomnia, hallucinations, anxiety; in evolution: cognitive slowing, flat affect, akathisia	12 h video EEG: normal	Brain CT: normal; Brain MRI (native and with gadolinium): unremarkable	Normal	N	Antineural autoantibodies evidenced in CSF by immunostaining	IVIg (2 g/kg, over 3 days)	Good evolution, regression of the symptoms

Table abbreviations: M = Male; F = Female; D = Days; P = Positive; N = Negative; NA = Not Available.

## Data Availability

Available upon request.
